# Delirium in Medically Hospitalized Patients: Prevalence, Recognition and Risk Factors: A Prospective Cohort Study

**DOI:** 10.3390/jcm12123897

**Published:** 2023-06-07

**Authors:** Rajaa Saleh Al Farsi, Abdullah M. Al Alawi, Aisha Ramadhan Al Huraizi, Taif Al-Saadi, Noof Al-Hamadani, Khalfan Al Zeedy, Juhaina Salim Al-Maqbali

**Affiliations:** 1 Oman Medical Specialty Board, Internal Medicine Residency Training Program, Muscat 130, Oman; rajaa-alfarsi@hotmail.com; 2 Department of Medicine, Sultan Qaboos University Hospital, Muscat 123, Oman; a.alhuraizi@squ.edu.om (A.R.A.H.); khalfanalzidii@gmail.com (K.A.Z.); 3 School of Medicine, Royal College of Surgeons in Ireland, D02 YN77 Dublin, Ireland; taif.alsaadi@hotmail.com (T.A.-S.); noof.alhamadani@hotmail.com (N.A.-H.); 4 Department of Pharmacy, Sultan Qaboos University Hospital, Muscat 123, Oman; juhaina@squ.edu.om; 5 Department of Pharmacology and Clinical Pharmacy, College of Medicine and Health Science, Sultan Qaboos University, Muscat 123, Oman

**Keywords:** delirium, prevalence, incidence, elderly, risk factors

## Abstract

Background: Delirium is a common neuropsychiatric syndrome in hospitalized elderly patients and is associated with poor clinical outcomes. We aimed to determine the prevalence, recognition, risk factors, and course of delirium among hospitalized elderly (65 years of age or older) patients at Sultan Qaboos University Hospital (SQUH). Methods: A prospective cohort study included 327 elderly patients (65 years of age or older) admitted to the medical wards at SQUH. Patients were screened for delirium using the 3-Minute Diagnostic Confusion Assessment Method (3D-CAM). Additionally, medical records were reviewed to identify possible associated factors. Results: The prevalence of delirium was 55.4% (95% CI 49.9–60.7), and 35.4% of patients with delirium were not recognized by the treating team. Hypoactive delirium is the most common type of delirium. The logistic regression analyzes demonstrated that pre-existing cognitive impairment (OR = 4.0); poor functional status (OR = 1.9); the use of medications that are known to precipitate delirium (OR = 2.3); polypharmacy (OR = 5.7); urinary catheterization (OR = 2.2); dehydration (OR = 3.1); and electrolytes derangements (OR = 2.0) were independent risk factors for delirium. Furthermore, 56.9% of patients with delirium continued to have delirium upon discharge from the hospital. Conclusions: Delirium is common among elderly patients hospitalized in general medical wards. Implementing effective preventive strategies for delirium during the hospital stay, including early recognition using standard sensitive and specific screening tools (i.e., 3D-CAM) and developing geriatric wards, is crucial.

## 1. Introduction

Delirium is a neuropsychiatric syndrome characterized by an acute onset of altered consciousness, cognitive impairment, and inattention that fluctuates in severity over time [1]. Delirium is usually reversible by treating the causative condition. Unlike dementia, delirium is acute in onset, has a fluctuating course, and is typically reversible [2].

Delirium is more common in the elderly and can be the only presentation of many serious medical conditions in this age group [3]. A recent systematic review showed that delirium is present in approximately 50% of hospitalized elderly patients (aged 65 years or older) [4]. Moreover, 15–25% of the elderly develop delirium after major elective surgery, and the incidence can reach 50% after high-risk procedures such as hip fracture repair and cardiac surgery [5,6]. Furthermore, delirium prevalence is as high as 80% among patients of any age admitted to the intensive care unit (ICU) requiring mechanical ventilatory support [7].

Although delirium is a common condition in hospitalized patients, it is still often not recognized and is poorly managed in clinical practice. This can be explained by different clinical presentations, fluctuating symptoms, the presence of delirium in patients with pre-existing dementia, and the lack of routine cognitive assessment in the hospital [8]. Early recognition of delirium in acute health settings can improve patient health outcomes, reduce hospital stays, and prevent unnecessary healthcare costs [1].

Several studies were conducted to measure the prevalence and incidence rates of delirium in hospitalized elderly patients in medical wards. However, there are discrepancies in the reported prevalence, risk factors, and course of delirium. This study aimed to determine the prevalence and recognition of delirium among medically hospitalized elderly (65 years or older) patients in a tertiary healthcare setting using validated screening tools. Furthermore, we aimed to identify the risk factors, type, and course of delirium.

## 2. Methods

### 2.1. Study Population and Design

The study was a prospective cohort study conducted at Sultan Qaboos University Hospital (SQUH). SQUH is a 600-bed multispecialty-teaching tertiary hospital that provides care for parts of the Muscat and Al-Batinah governorates. It is the main referral center for all regional hospitals in Oman [9,10,11]. The study included all patients aged 65 years and older admitted to acute medical ward units from 1 January 2022 to 31 May 2022. Patients admitted directly to the intensive care unit, the coronary care unit, and the hematology and oncology wards were excluded. In addition, patients with language barriers or aphasia were excluded from the study.

### 2.2. Sample Size

Based on the previously estimated prevalence of delirium in elderly patients of 30% in similar healthcare settings [1,12] and an average length of hospital stay duration of five days in our hospital setting [11], we calculated that we needed 320 patients (95% confidence interval and 5% error margin).

### 2.3. Assessment Tools and Data Collection

Prospective data collection involved conducting interviews with patients and their relatives, as well as reviewing electronic medical records to gather pertinent clinical and laboratory data related to the current hospital visit. All enrolled patients were screened for delirium by trained research assistants (medical and nursing trainees) within 24 h after admission, every two days in the first week, and then weekly until they developed delirium or were discharged from the hospital. Additionally, patients’ electronic medical records and nursing notes were evaluated daily, and patients were screened for delirium if clinical notes indicated possible symptoms of delirium (e.g., confusion, agitation, disorientation, etc.).

The 3-Minute Diagnostic Confusion Assessment Method (3D-CAM) was used to screen patients for delirium. The 3D-CAM is a validated bedside measurement tool used for the detection and diagnoses of delirium with (94%) sensitivity and (89%) specificity [13]. It includes objective measurements and clinical observation to determine the presence of delirium. The 3D-CAM assesses the presence of four characteristics of delirium: acute onset or fluctuating course (feature 1), inattention (feature 2), disorganized thinking (feature 3), and altered level of consciousness (feature 4). The diagnosis of delirium requires the presence of features 1 and 2 of 3D-CAM and either 3 or 4 [14]. We used the Arabic version of 3D-CAM, which was previously validated in a similar healthcare setting [15].

For all patients who developed delirium during hospitalization, additional data were collected, including the following: date of onset of delirium; type of delirium (hyperactive, hypoactive, or mixed); in-hospital course of delirium: transient (that is, recovered within 24 h), recovered (that is, recovered by discharge), or persistent (that is, delirium present at discharge); the cause of delirium; the medication used to control agitation in patients with delirium; and recognition of delirium by the treating team (that is, documentation of delirium or a term indicated delirium in the medical record before delirium identified by the research team).

In addition, all included patients were screened for pre-existing cognitive impairment and possible dementia using the Informant Questionnaire on Cognitive Decline in the Elderly Short Form (IQCODE-SF). IQCODE-SF is a validated screening tool for detecting cognitive impairment that is also used to detect dementia. It includes 16 items asked of the patient’s relative that retrospectively assess the change in cognitive and functional performance over 10 years. For each item, they have to rate the change from 1 to 5, where higher scores indicate a greater degree of cognitive decline. The average final score was 1.0 to 5.0 [16,17], where the cut-off score suggestive of dementia defined in previous studies ranged from 3.3 to 3.6 [16].

In a systematic review published in 2015, it was found that using a cut-off point of 3.3 in a secondary-care setting had a sensitivity of 91% (95% CI 0.86 to 0.94) and a specificity of 66% (95% CI 0.56 to 0.75) for diagnosing dementia [16]. Another study conducted in the Arabic population utilized the Arabic form of IQCODE and found that using a cut-off point > 3.34 had a sensitivity of 92.5% and specificity of 94.4% for dementia screening [17]. Based on these findings, we opted to use a cut-off point of 3.5 to define possible dementia in patients who did not have a prior diagnosis of dementia before or during their hospital stay.

The Katz Index of Independence in Basic Activities of Daily Living (Katz ADL) was used to estimate the pre-morbid functional status of the patient one month before hospitalization. Katz ADL assesses whether the patient can independently perform six primary functions: bathing, dressing, toileting, transferring, continence, and feeding. A score of 6 reflects full function; 3 to 5 reflects moderate functional impairment; and 2 indicates the presence of severe functional impairment [18]. In addition, we collected other relevant demographic, clinical, and biochemical data. We included data on possible risk factors for delirium, including the following polypharmacy (defined as current use of 5 medications) [19]; the presence of comorbidities (such as dehydration, anemia, electrolyte imbalance, hyponatremia, hypernatremia, hypercalcemia, hypocalcemia, hypokalemia, hyperkalemia, hypomagnesemia); organ failure (that is, decompensated heart failure, acute kidney injury, acute liver failure/or chronic liver disease, and acute respiratory failure); presence of a urinary catheter; use of non-invasive mechanical ventilation (NIV); and use of medication that can increase risk of delirium, including benzodiazepines, opioids, anticholinergics, antidepressants, antihistamines, antipsychotic, dopamine agonist, steroids, or diuretics.

Finally, we labeled the recognition of delirium by the treating team as recognized delirium considering any source of documentation performed by a physician or nurse who took care of the patient to indicate the presence of delirium at any time before the assessment by the members of the research team.

### 2.4. Statistical Analysis

Categorical variables were reported as numbers and percentages, while continuous variables were expressed as means for normally distributed data or median (IQR) for abnormally distributed data. Continuous variables between the two groups were compared using the student *t*-test for normally distributed variables or the Wilcoxon rank sum for abnormally distributed variables. The chi-squared test was used to assess the association between categorical variables.

All relevant independent factors were included in the stepwise regression analysis backward to predictors of delirium. Hazard and odds ratios were calculated with 95% confidence intervals (CI). Two-sided *p*-values < 0.05 were considered to be statistically significant. Statistical calculations were performed using Stata v. 17 software package (StataCorp LLC, College Station, TX, USA).

### 2.5. Ethical Approval

The study was approved by the Medical Research Ethics Committee of the College of Medicine and Health Sciences of Sultan Qaboos University (REF. NO. SQU-EC/389/2021. MREC #2444). Before participation, informed consent was obtained from patients or their next of kin (if their capacity was impaired).

### 2.6. Funding Statement

The study received a grant from the Ministry of Education, Research, and Innovation, Oman (BFP/GRG/HSS/21/023).

## 3. Results

**Figure 1:**
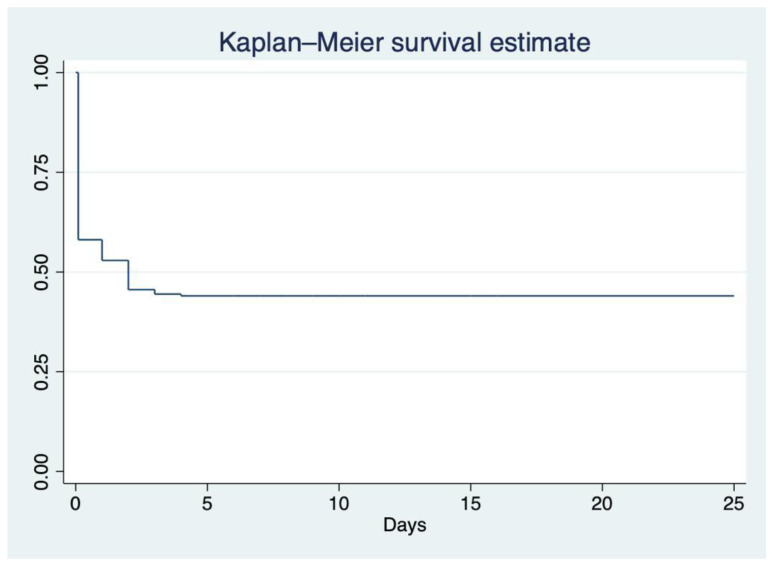
Kaplan–Meier survival analysis demonstrates the time between admission and diagnosis of delirium (n = 327).

During the study period, 455 patients (65 years of age or older) were admitted to the medical wards, and 327 met the inclusion criteria. The mean age of the study population was 71 years (IQR: 66–78 years), and 50.5% (n = 165) were men. The prevalence of delirium was 55.4% (n = 181, 95% CI 49.9–60.7). Most patients with delirium (n = 181) developed delirium early on admission, within 72 h after admission (Figure 1).

Among patients with delirium, 33.2% (n = 64) were not recognized by the treating team members. Univariate analysis showed that old age (74.5 vs. 68 years; *p* < 0.01); COVID-19 infection (8.8% vs. 2.7%; *p* = 0.02); pre-existing cognitive function (35.4% vs. 7.5%; *p* < 0.01); impaired functional ability (74.6% vs. 42.5%; *p* < 0.01); physical restrain (3.3% vs. zero; *p* = 0.035); anemia (67.4% vs. 47.9%; *p* < 0.01); urinary catheterization (66.2% vs. 31.5%; *p* < 0.01); dehydration (71.3% vs. 33.5%; *p* < 0.01); impaired vision or/and hearing (37.0% vs. 23.3%; *p* < 0.01); organ failure (69.1% vs. 58.2%; *p* = 0.030); electrolytes disturbances (84.0% vs. 60.3%; *p* < 0.01); the presence of ≥ 3 morbidities (90.6% vs. 78.7%; *p* < 0.01); the use of medications known to precipitate delirium (89.0% vs. 72.6%; *p* < 0.01); and polypharmacy (97.8% vs. 88.4%; *p* < 0.01) were more common among hospitalized patients who developed delirium (Table 1).

Stepwise backward regression demonstrated that pre-existing cognitive impairment (OR = 4.0); poor functional status (OR = 1.9); the use of medications known to precipitate delirium (OR = 2.3); polypharmacy (OR = 5.7); urinary catheter (OR = 2.2); dehydration (OR = 3.1); and electrolytes derangements (OR = 2.0) were independent risk factors for delirium (Table 2).

The primary diagnoses classified according to ICD-10 did not differ between the patients who developed delirium and those without delirium (Table 3).

Hypoactive delirium was common (65.2%, n = 118) compared to other types of delirium. Only 28.2% of the patients (n = 51) received medications for delirium, and benzodiazepines were the most commonly prescribed medication for delirium. In terms of the progression of delirium, a majority of patients (56.9%, n = 103) still exhibited signs and symptoms of delirium even after being discharged from the hospital, as confirmed by follow-up phone calls (Table 4). A time-to-event analysis demonstrated that delirium occurred early on admission (Figure 1).

## 4. Discussion

This study used validated tools to prospectively assess delirium in acute care wards in a tertiary care setting and included patients with COVID-19 infection. The prevalence of delirium was high (55.4%); delirium was not recognized in one-third of patients with delirium, and two-thirds continued to have delirium upon hospital discharge. Hypoactive delirium is the most common type of delirium. Furthermore, pre-existing cognitive impairment, poor functional status, medications known to cause delirium, polypharmacy, urinary catheters, dehydration, and electrolyte derangements were independent risk factors for delirium.

According to the findings of this study, the prevalence of delirium among elderly patients (65 years old) admitted to the medical wards is 55.4%. This rate is higher compared to the prevalence reported in other studies worldwide, which ranged from 17.9% to 40.4% [15,20,21,22]. This can be explained by variations in the baseline characteristics of patients, study settings, and methods used to identify delirium. We have included an older population compared to a previous study from Saudi Arabia, which explained the higher prevalence of delirium in our setting (55.4% vs. 21.8%) [15]. In addition, a significant number of our cohort had pre-existing cognitive impairment, poor baseline ADL, polypharmacy, and multiple comorbidities, which are known risk factors for delirium. Furthermore, the current study was conducted in a tertiary hospital where most of the hospitalized patients were critically ill. Furthermore, the design of the medical ward (crowded, with a lack of natural light) in our healthcare setting may contribute to the high prevalence of delirium. The noisy hospital environment, alarms, lack of natural light, lack of views of the outside environment, and views of nature have been linked to delirium [23,24]. Our study demonstrated that almost all patients with delirium developed delirium early on admission, which is similar to the findings of previous studies [25]. Therefore, preventive measures against delirium should start early upon hospitalization [26].

We showed that elderly patients with pre-existing cognitive impairment or possible dementia have a four-times higher risk of developing delirium during hospitalization (OR 4, *p* < 0.01; 95% CI 1.8–9.0), which aligns with other studies [15,27,28]. The higher prevalence of delirium in patients with dementia or pre-existing cognitive impairment was expected, given the advanced age of these individuals. It is crucial to acknowledge that delirium is a multifactorial condition influenced by a variety of complex factors associated with the aging process [27,28,29].

We also showed that delirium was common in elderly patients admitted with COVID-19 infection; 16 out of 20 patients (80%) developed delirium during their hospital stay. However, we could not estimate the prevalence, as the study included a small number of patients with COVID-19 infection. According to a systematic review and meta-analysis, which included 48 studies from 13 countries, the prevalence of delirium in patients with COVID-19 infection ranged from 2.8% to 77.4%. The pooled analysis showed that the prevalence of delirium in patients 65 years of age or older and with COVID-19 infection was 28.2% (95% CI: 23.5–33.1%) [30]. Multiple studies have suggested that the pathophysiology of delirium in patients with COVID-19 infection is multifactorial. Some evidence suggests a direct effect of Coronavirus 2 from Severe Acute Respiratory Syndrome Coronavirus 2 (SARS-CoV-2) on the central nervous system (CNS). Furthermore, the complications of COVID-19 infection, such as pneumonia and acute respiratory distress syndrome (ARDS), which result in hypoxia, respiratory failure, and other organ failures, are all risk factors for delirium, which can also result in hypoxic-ischemic encephalopathy and uremic encephalopathy in the case of acute renal injury (AKI) [31]. In severe COVID-19 infections, the systemic inflammatory response can increase the permeability of the blood–brain barrier (BBB) and allow released cytokines such as TNF-α, IL-1, and IL-6 to enter the brain and cause neuronal damage [32,33]. Furthermore, the medications used in the management of COVID-19 infection, such as steroids and sedatives, can be associated with an increased risk of delirium. Furthermore, isolation as part of COVID-19 precautions can limit the support and orientation of patients’ family members. Wearing personal protective equipment (PPE) may be a reason for older patients’ disorientation [31,33].

Anemia was found to be significantly associated with delirium. Previous studies showed similar findings [34]. There is no clear explanation for the pathophysiology of delirium in patients with anemia. However, a possible hypothesis could be that anemia results in impaired oxygen delivery to the brain, which results in delirium. Furthermore, patients with anemia due to hemorrhagic shock and hypotension result in hypoperfusion of the brain, which causes delirium. Furthermore, few studies have shown a significant association between anemia and cognitive impairment, dementia, and an increased risk of delirium [35,36]. Furthermore, anemia in the elderly leads to a decrease in physical performance and quality of life [37]. However, a systemic review of 23 studies showed a lack of good-quality evidence supporting blood transfusion as a preventive measure against delirium in patients with anemia [38].

The study findings showed a significant association between the use of urinary catheterization and delirium, which aligns with previous studies [27,39,40,41]. Using urinary catheterization increases the risk of urinary tract infections and urosepsis and restricts patient mobility. All these factors increase the risk of delirium [42]. These findings support the importance of the early removal of the urinary catheter to prevent delirium.

The current study showed that the prevalence of delirium was slightly higher in women than in men (58% and 52%, respectively), which is similar to the findings of previous studies [43,44]. However, a previous systematic review did not show a significant association between gender and delirium [27]. Moreover, the study findings showed that constipation, the use of non-invasive mechanical ventilation, depression, and sleep deprivation were higher in patients with delirium; however, there was no significant association.

The primary treatment for delirium is to identify and manage the reversible factors. Drugs are the most common reversible factors that cause delirium. Polypharmacy, defined as the chronic use of five medications or more [45], is significantly associated with the prevalence of delirium in our cohort, which aligns with other observational studies [45,46,47,48], in which polypharmacy was more common in elderly patients with comorbidities [47,49]. Furthermore, polypharmacy has been associated with a higher incidence of adverse side effects, an increased risk of hospitalization, and mortality [50,51]. Observational studies show that the most common drugs associated with delirium are sedative–hypnotics (benzodiazepines), analgesics (narcotics), and medications with an anticholinergic effect [52]. Elderly patients are more prone to drug-related delirium due to multiple comorbidities, polypharmacy, drug–drug interactions, and physiological changes with aging, which all alter the pharmacokinetics and pharmacodynamics of these drugs [53].

In the current study, only 28.2% of the patients received medications to control their symptoms of delirium; benzodiazepines were the most prescribed medications for delirium, which is not in line with the practice of updated evidence. According to a recent systematic review, there is not enough sufficient evidence to support the use of antipsychotic medication to prevent delirium [54]; therefore, the routine administration of antipsychotic medication is not recommended to prevent delirium. However, it can treat agitated delirium when non-pharmacological measures do not control symptoms [55,56,57]. Moreover, it should be used cautiously for the shortest duration at the lowest effective doses. It may prolong delirium and increase the risk of complications, including the risk of aspiration and falls [8].

Benzodiazepines are generally avoided by hospitalized patients with delirium. They have been shown to be associated with worse outcomes and are an independent risk factor for delirium. However, in delirium secondary to alcohol withdrawal, benzodiazepines are the recommended therapy [58]. Haloperidol was the most common antipsychotic used to control symptoms of delirium; recently, atypical antipsychotic drugs (i.e., quetiapine) have emerged in the treatment of delirium, which have better safety profiles, including the lowest extrapyramidal side effect [58,59]. Overall, the evidence encourages multicomponent delirium prevention and treatment strategies with non-pharmacological intervention, including reorientation, early mobility, risk factor evaluation, modification (e.g., drugs and medical devices), nutrition support, normalization of the sleep–wake cycle (e.g., noise reduction), and integrating family into the care [55].

In the current study, 35.4% were not recognized by medical staff or recognized late. The reason for this is the lack of screening tools to diagnose delirium in our healthcare setting. Most elderly patients tend to have hypoactive delirium, as evidenced in the current study, which may go unrecognized in the absence of routine delirium screening tools in elderly patients. These findings emphasize the need for a screening tool to diagnose delirium, especially in at-risk patients.

This study is one of the few studies in the Middle East region that assesses delirium in medically hospitalized patients in tertiary healthcare settings. The findings of this study provide important data and insights for healthcare system managers and stakeholders on the prevalence, risk factors, and recognition of delirium, which will influence the need to implement protocols and guidelines for the screening and prevention of delirium in hospitalized elderly patients. In addition, it emphasizes the need to establish a geriatric ward. Furthermore, the result of this study can be used as a baseline and data source for further research on interventional trials for the prevention and treatment of delirium in our healthcare setting. Our study has limitations that should be addressed. Firstly, conducting the study at a single center limits the generalizability of our findings to other healthcare settings. In addition, including patients exclusively from a tertiary hospital introduces the potential for selection bias, restricting the applicability of our results to a broader patient population. Additionally, the multifactorial nature of delirium poses challenges for identifying a single underlying cause. Our study relied on questionnaires administered by patients’ relatives to detect cognitive impairment and possible dementia. While this approach has merits, it introduces the possibility of information bias due to reliance on subjective reports. Furthermore, excluding patients admitted directly to specialized units such as intensive care, coronary care, hematology wards, and oncology wards limits our understanding of the prevalence and characteristics of delirium in critically ill individuals and those with specific medical conditions. Consequently, the generalizability of our findings to these patient groups is limited.

## 5. Conclusions

This study provides important insights into the assessment of delirium among medically hospitalized patients in a tertiary healthcare setting, including those with COVID-19 infections. These findings, including a high prevalence of delirium and a predominance of hypoactive delirium, have significant clinical implications. The identified risk factors, such as pre-existing cognitive impairment, poor functional status, medications associated with delirium, polypharmacy, urinary catheter use, dehydration, and electrolyte imbalances, highlight the multifactorial nature of delirium. Several strategies can be considered to enhance the translation of our findings to other healthcare settings, including implementing standardized delirium screening protocols, fostering interprofessional collaboration, and improving the medical ward setting. This can involve the establishment of dedicated geriatric wards to provide specialized care for older patients, addressing their unique needs, and potentially reducing the occurrence and impact of delirium. By implementing these strategies, healthcare systems can enhance delirium identification, understanding, and care, ultimately improving patient outcomes. Proactive measures that address the multifactorial nature of delirium and improvements in the medical ward setting are crucial for reducing the burden associated with this complex condition and optimizing patient well-being.

## Figures and Tables

**Table 1:** Demographics of the patients and risk factors associated with delirium (total n = 327). Bold: Risk factors for delirium.

Characteristic, n (%) Unless Specified Otherwise	Total (n = 327)	No Delirium (n = 146)	Delirium (n = 181)	*p*
Age (IQR), years	71 (66–78)	68 (64–74)	74.5 (67–80)	**<0.01**
Men (n)	165 (50.5%)	78 (53.4%)	87 (48.1%)	0.261
Risk factors				
COVID-19	20 (6.1%)	4 (2.7%)	16 (8.8%)	0.02
Pre-existing cognitive function	75 (22.94%)	11 (7.5%)	64 (35.4%)	**<0.01**
Impaired functional ability	197 (60.2%)	62 (42.5%)	135 (74.6%)	**<0.01**
Physicals restrain	6 (1.8%)	0	6 (3.3%)	**0.035**
Depression	18 (5.5%)	7 (4.8%)	11 (6.1%)	0.595
History of drug and alcohol abuse	14 (4.3%)	8 (5.5%)	6 (3.3%)	0.349
History of surgery or anesthesia during admission	77 (23.7%)	30 (20.5%)	47 (26.0%)	0.229
Anemia	192 (58.9%)	70 (47.9%)	122 (67.4%)	**<0.01**
Urinary catheterization	166 (50.8%)	46 (31.5%)	120 (66.2%)	**<0.01**
Dehydration	178 (54.4%)	49 (33.5%)	129 (71.3%)	**<0.01**
Constipation	132 (40.50%)	53 (36.3%)	79 (43.6%)	0.165
Impaired sensation (e.g., vision and hearing)	101 (31.2%)	34 (23.3%)	67 (37.0%)	**<0.01**
Mechanical ventilation	82 (25.1%)	34 (23.3%)	48 (26.5%)	0.463
Pain	69 (21.2%)	33 (22.6%)	36 (19.9%)	0.585
Organ failure	210 (65.0%)	85 (58.2%)	125 (69.1%)	**0.030**
Electrolytes disturbances	240 (73.6%)	88 (60.3%)	152 (84.0%)	**<0.01**
History of sleep deprivation	35 (10.7%)	11 (7.5%)	24 (13.3%)	0.086
Multiple comorbidities (≥3)	279 (85.3%)	115 (78.7%)	164 (90.6%)	**<0.01**
Use of medications known to precipitate delirium	267 (81.9%)	106 (72.6%)	161 (89.0%)	**<0.01**
Polypharmacy (≥5)	306 (93.6%)	129 (88.4%)	177 (97.8%)	**<0.01**

**Table 2:** Logistic regression analysis: factors associated with delirium in medically hospitalized patients (n = 327).

Factor	Odd Ratio (OR)	*p* Value	95% CI
Pre-existing cognitive impairment	4.0	<0.01	1.8–9.0
Poor functional ability	1.9	0.02	1.1–3.4
Use of any medications known to precipitate delirium	2.3	0.04	1.1–4.9
Polypharmacy	5.7	0.03	1.1–28.2
Urinary catheter	2.2	<0.01	1.3–3.8
Dehydration	3.1	<0.01	1.8–5.3
Electrolytes derangement	2.0	0.03	1.1–3.8

**Table 3:** Primary diagnoses classified according to ICD-10 in patients with and without delirium.

Primary Diagnosis (ICD-10)	Total (n = 327)	No Delirium (n = 146)	Delirium (n = 181)	*p*
Infectious disease (A00-B99)	46 (14.07%)	15 (10.27%)	31 (17.13%)	0.076
Hematological diseases (D50-D89)	6 (1.83%)	2 (1.37%)	4 (2.21%)	0.695
Endocrine, nutritional, and metabolic diseases (E00-E90)	35 (10.70%)	19 (13.01%)	16 (8.84%)	0.253
Diseases of the nervous system (G00-G99)	41 (12.54%)	18 (12.33%)	23 (12.71%)	0.918
Diseases of the circulatory system (I00-I99)	55 (16.82%)	28 (19.18%)	27 (14.92%)	0.918
Diseases of the respiratory system (J00-J99)	67 (20.49%)	28 (19.18%)	39 (21.55%)	0.598
Diseases of the digestive system (K00-K93)	29 (8.87%)	14 (9.59%)	15 (8.29%)	0.681
Diseases of the genitourinary system (N00-N99)	16 (4.89%)	9 (6.16%)	7 (3.87%)	0.338
Others	32 (9.79%)	13 (8.90%)	19 (10.50%)	0.630

**Table 4:** Recognition, course, and treatment of delirium (n = 181).

Delirium Recognition	117 (66.48%)
***Type of Delirium***	
Hypoactive	118 (65.2%)
Hyperactive	13 (7.2%)
Mixed	50 (27.6%)
***Treatment***	
**Medication used for Delirium**	51 (28.18%)
Benzodiazepines	37 (20.44%)
Atypical antipsychotic	13 (7.18%)
Typical antipsychotic	6 (3.31%)
Tricyclic antidepressant	6 (3.31%)
Physical restrain	10 (5.52%)
***Course of Delirium***	
Transit (resolved within 24 h)	31 (17.1%)
Resolved prior to hospital discharge	47 (26.0%)
Persist	103 (56.9%)

## Data Availability

Data are available on request from the corresponding author.
